# School Medical Inspection and the War: The Part of the Medical Inspector

**Published:** 1915-03-27

**Authors:** 


### SCHOOL MEDICAL INSPECTION AND THE WAR.

### The Part of the Medical Inspector.

During this time of stress and trouble, when all the ordinary routine of daily professional life is curtailed, it seems strange to some people that the duties of the school medical inspectors have not been similarly curtailed. Yet it must be admitted that these officials are doing a national work, and that the effects of their efforts, if not immediately apparent, may be of considerable value to the nation before the war is over.

### Diseases of Compulsory Education.

When we cast a glance at the returns of rejections for physical defects in recruits, we are struck by two or three outstanding facts. One is that the vast majority of these defects are defects which could and should have been cured or remedied during childhood; another is that some of these defects are due to occupational and environmental causes. In certainly a percentage of the latter class the original foundation for the defect was laid at school, or at least during the child's school-going age. Lateral curvature, flat-foot, and myopia are pre-eminently diseases of a generation that has compulsory education; the faulty positions adopted at school are factors in evolving all three. Not factors that work by themselves; they nearly always have a suitable soil in hereditary or congenital weakness in which to work, but they are developed by constant assumption of faulty attitudes which ignorant or careless teachers have not the gumption to correct. For many years we have been blaming the desks and school furniture, but more recently we have come to the conclusion, amply warranted by the data collected from laborious investigations, that while school furniture plays a part in the causation of these postural defects, the chief part is played, undoubtedly, by fatigue due to a variety of causes. Myopia by itself is probably a minor defect in an ordinary child; in a child who occupies a faulty position in school, in a bad light, who uses bad print and whose eyes have to contend against glare and reflection from walls and blackboard and glazed book pages, it may soon become, and generally does become, a very serious defect. Similarly, flat-foot, which in ordinary life may entail little besides temporary inconvenience to its victim, becomes a very serious defect in a soldier who has to march far and hard.

### Preventive Work.

The school doctor's task is essentially to eliminate these defects partly by curing or remedying those already in existence, but more largely by preventing them in future generations. By so doing he is accomplishing a national work of the greatest importance, for the elimination of these defects indubitably raises the health and physical status of the adulthood of the community and consequently diminishes the percentage of the unfit. Similarly the school doctor's work lies in safeguarding the children of the State from the effects of disease that at present exacts a very heavy toll, not indeed in death, but in chronic invalidity. Measles, scarlet fever, and even rheumatic fever are in themselves comparatively unimportant diseases in so far as they influence the vital statistics of the population between the ages of five and fourteen. But when one considers their effects upon the community as a whole, one realises to the full that they are exceedingly important and serious elements in undermining the physique and the health of the nation.

### Defects of Recruits.

These three diseases are undoubtedly primarily responsible for the vast majority of heart lesions that cause so many recruits to be rejected at the medical examinations. Rheumatic fever is the chief factor in causing valvular disease in early youth, as most doctors will readily admit, but the responsibility of measles and scarlet fever is not yet so conclusively demonstrable. Yet it seems clear that most of the rejections for "disorganised action of the heart"—the familiar D.A.H. of the returns—are the result of one of these two diseases of childhood plus fatigue and strain of some kind. The school doctor's duty lies therefore in striving to control the after-effects of these diseases, and by inculcating in teachers and parents the truth with regard to the seriousness of these sequelæ, to bring about a better condition of things whereby less strain is thrown upon the school-going child.

It would be interesting to find out how many of the elder children who have already passed the doctors attached to the schools have been before the recruiting doctors and passed these latter. The records of these children are still, doubtless, available, and it would be interesting to collate the findings at the medical inspection, say six years ago, with the findings of to-day. Especially interesting would the results be in regard to heart lesions.

### The War and Research.

Six years ago, school doctors were undoubtedly more concerned about transient bruits and murmurs in the hearts of school children than they are now, and were inclined—wrongly, in our opinion—to attach too much weight to these findings as demonstrating some organic lesion. Experience shows that many of the children with such transient murmurs, and some even with what at first hearing appear to be real organic murmurs, do excellently well in later life, and we should not be surprised if many of these boys have successfully passed the doctor and are now equally successfully bearing their share of the hard work in the trenches. We commend this work to the attention of school doctors who are wishful of some subject for a special note; the results of such an investigation as we have suggested will not only be interesting but instructive as well.

